# Tolerance to Proinsulin-1 Reduces Autoimmune Diabetes in NOD Mice

**DOI:** 10.3389/fimmu.2021.645817

**Published:** 2021-03-25

**Authors:** Gaurang Jhala, Claudia Selck, Jonathan Chee, Chun-Ting J. Kwong, Evan G. Pappas, Helen E. Thomas, Thomas W.H. Kay, Balasubramanian Krishnamurthy

**Affiliations:** ^1^ St. Vincent’s Institute, Fitzroy, VIC, Australia; ^2^ Department of Medicine, The University of Melbourne, St Vincent’s Hospital, Fitzroy, VIC, Australia; ^3^ National Centre for Asbestos Related Diseases, Institute of Respiratory Health, University of Western Australia, Perth, WA, Australia; ^4^ School of Biomedical Sciences, University of Western Australia, Perth, WA, Australia

**Keywords:** type 1 diabetes, proinsulin-1, CD4+ T cells, immune tolerance, NOD mice

## Abstract

T-cell responses to insulin and its precursor proinsulin are central to islet autoimmunity in humans and non-obese diabetic (NOD) mice that spontaneously develop autoimmune diabetes. Mice have two proinsulin genes proinsulin -1 and 2 that are differentially expressed, with predominant proinsulin-2 expression in the thymus and proinsulin-1 in islet beta-cells. In contrast to proinsulin-2, proinsulin-1 knockout NOD mice are protected from autoimmune diabetes. This indicates that proinsulin-1 epitopes in beta-cells maybe preferentially targeted by autoreactive T cells. To study the contribution of proinsulin-1 reactive T cells in autoimmune diabetes, we generated transgenic NOD mice with tetracycline-regulated expression of proinsulin-1 in antigen presenting cells (TIP-1 mice) with an aim to induce immune tolerance. TIP-1 mice displayed a significantly reduced incidence of spontaneous diabetes, which was associated with reduced severity of insulitis and insulin autoantibody development. Antigen experienced proinsulin specific T cells were significantly reduced in in TIP-1 mice indicating immune tolerance. Moreover, T cells from TIP-1 mice expressing proinsulin-1 transferred diabetes at a significantly reduced frequency. However, proinsulin-1 expression in APCs had minimal impact on the immune responses to the downstream antigen islet-specific glucose-6-phosphatase catalytic subunit-related protein (IGRP) and did not prevent diabetes in NOD 8.3 mice with a pre-existing repertoire of IGRP reactive T cells. Thus, boosting immune tolerance to proinsulin-1 partially prevents islet-autoimmunity. This study further extends the previously established role of proinsulin-1 epitopes in autoimmune diabetes in NOD mice.

## Introduction

Recognition of proinsulin by the immune system is a major determinant in the pathogenesis of autoimmune diabetes in both humans and non-obese diabetic (NOD) mice (1, 2). A polymorphic variable number of tandem repeats (VNTR) located in the promoter region of the insulin locus controls the transcription level of the *Ins* gene and is strongly associated with susceptibility to type 1 diabetes (T1D) in humans (3–5). Mice do not have a VNTR upstream of the insulin locus; however, they have two Insulin genes, *Ins1* and *Ins2* encoding proteins that are highly homologous with 92% identity at the amino acid level. Proinsulin 1 and 2 proteins have identical A chains but differ by two amino acids in the B chain, three amino acids in the connecting peptide (C-peptide) and six amino acids in the leader peptide (6). The two proinsulin isoforms are differentially expressed with proinsulin 1 (PIns1) predominantly expressed in the pancreatic beta-cells and proinsulin 2 (PIns2) being the predominant isoform detected in the thymus (7–9).

Immune responses to native insulin peptides, in particular the B chain amino acids 9-23 (Ins B:9-23), are essential for autoimmune diabetes in NOD mice (10, 11). The two proinsulin isoforms differ by a single amino acid in the B: 9-23 region (PIns1: B9 proline, PIns2: B9 serine) and strong cross-reactivity of T cells for the Ins B: 9-23 epitope in both proinsulin molecules has also been reported (12). Despite the high degree of homology in the B:9-23 epitope and cross-reactivity of T cells for the Ins B: 9-23 epitope, a divergent immune response was observed when NOD mice were immunized with either Ins1 B:9-23 or Ins2 B:9-23 peptides, with Ins2 peptide conferring protection from diabetes onset, whereas Ins1 peptide did not prevent disease (13, 14). Further differences in cellular and humoral immune responses to both proinsulin isoforms have been highlighted by individual gene knockouts. NOD mice lacking *Ins2* gene develop accelerated diabetes, ascribed to loss of central tolerance to insulin peptides; however, development of insulin autoantibodies (IAA) in *Ins2* -/- mice suggests that immune responses against PIns1 epitopes are intact (15). In contrast, genetic deletion of *Ins1* or replacement of murine *Ins1* with human insulin gene (*INS*) in NOD mice provides significant protection from diabetes (16, 17). Protection from diabetes in NOD mice lacking *Ins1* is likely due to the absence of cognate antigen in the target tissue, indicating that PIns1 peptides may be primarily targeted by insulin reactive T cells. Immunogenic epitopes in the PIns1 molecule have been reported (18), and T cells recognizing PIns1 amino acids 47-64 in the C-peptide region induce diabetes in NOD.SCID recipients (19). Thus, epitopes in PIns1 molecule may contribute to islet autoimmunity.

In contrast to NOD mice, non-autoimmune strains lacking *Ins2* globally (20), or in medullary thymic epithelial cells (mTECs) did not develop pathological islet destruction however, when C57Bl/6 mice lacking Ins2 in mTECs were crossed to *Ins1* knockout mice, the progeny developed spontaneous autoimmune diabetes within 3 weeks after birth (21). These studies suggest that thymic expression of PIns1 may add to the effect of PIns2 in eliminating insulin-specific autoreactive T cells. Constitutive or temporal expression of PIns2 in APCs induces recessive tolerance to PIns2 as it provides lasting protection from autoimmune diabetes in NOD mice (22). These mice were also thought to be tolerant to PIns1 epitopes because of cross-reactivity of the T cells to the conserved Ins B: 9-23 epitope. However, the role of PIns1 specific immune responses in pathogenesis of islet autoimmunity in NOD mice remains unclear, given the differential immune response observed upon immunization with Ins1 B:9-23 or Ins2 B:9-23 peptide. To resolve this, we investigated the impact of induced PIns1 expression in APCs on the development of antigen-specific T cells as well as insulitis and diabetes in NOD mice.

## Materials and Methods

### TetO-Ins1 Mice

To generate the TetO-Ins1 construct, a 411 bp cDNA fragment spanning the coding region of murine PIns1 was amplified by PCR using NOD pancreatic islet cDNA as a template and cloned into HindIII and EcoRV sites of the pTRE-tight plasmid (Clontech). A 1100 bp transgene cassette comprising of the TetO-minimal CMV promoter, followed by the PIns1 gene and a polyA signal was excised between Xho I sites and purified for injection into fertilized NOD/Lt ova using standard techniques. Founders and transgene positive offspring were screened by PCR using primers spanning the PIns1 gene (5’-TTAAGATATCTTCATTCATTATAGAACTC -3’) and the tetO-CMV promoter (5’-TCAGTGATAGAGAACGTATGTCG -3’).

### Other Mice

NOD/Lt mice were bred and housed at the bioresources center St. Vincent’s Hospital, Fitzroy. The NOD-IEα-tTA mice that drive the expression of tetracycline transactivator (tTA) under the control of MHC class II IEα promoter have been previously described (23) and were obtained from Prof. C. Benoist and Prof. D. Mathis (Dept of Pathology, Harvard Medical School, Boston, Massachusetts, USA). Generation of NOD8.3 mice, which express the TCRαβ rearrangements of the H-2Kd-restricted, β cell-reactive, CD8+ T cell clone NY8.3, was previously described in detail (24). TIP-1/8.3 mice were generated by crossing NOD-IEα-tTA-TetO-Ins1double transgenic TIP-1 mice with TCR transgenic NOD8.3 mice. All mice were bred, maintained and used under specific pathogen free conditions at St Vincent’s Institute (Melbourne, Australia). All experimental procedures followed the guidelines approved by the institutional animal ethics committee.

### Doxycycline Treatment

Untreated TIP-1 mice constitutively express proinsulin-1 in antigen presenting cells (APCs). To turn-off proinsulin-1 expression, doxycycline hyclate (Dox) (Sigma-Aldrich) was administered *via* drinking water at concentration of 2mg/ml. Water bottles were changed thrice weekly.

### RT-PCR

For total RNA extraction, whole spleen and thymus were harvested in cold Phosphate Buffered Saline (PBS). Tissue homogenates were prepared in RNA lysis buffer RA1 (Macherey-Nagel) from a 15mg slice of tissue using a tissue homogenizer. RNA was isolated using Nucleospin RNA II-isolate kits (Macherey-Nagel), and first strand cDNA was generated from 500ng RNA using High Capacity cDNA Reverse Transcription kits (Applied Biosystem) according to the manufacturers’ instructions. cDNA was diluted (1:20) and Real-time PCR analysis was performed using Rotor-Gene-RG-3000 cycler (Corbett Research, Sydney, Australia). Taqman gene expression primers murine insulin 1 (*Ins1*; Mm01950294_s1), murine β-actin (*Actb*; Mm00607939_s1) and murine Glyceraldehyde 3-phosphate dehydrogenase (*Gapdh*; Mm99999915_g1) were purchased from Applied Biosystems. To determine relative expression, Ct values of Insulin were subtracted from Ct values of reference genes for each sample and the difference (dCt) was plotted to determine the abundance of the gene of interest.

### Histology and Immunohistochemistry

For immunohistochemistry, pancreata were snap-frozen in optimal cutting temperature compound (OCT Compound; Sakura Finetek, Torrance, CA) and stored at −80°C. For histological analysis 5-µm frozen sections of pancreas were prepared from three levels (200 µm apart), acetone fixed, stained with guinea pig anti-insulin followed by horseradish peroxidase–conjugated anti–guinea pig Ig (Dako Cytomation, Carpenteria, CA) and counterstained with hematoxylin. Insulitis was graded using the following scale: 0 = no infiltrate, 1 = peri-islet infiltrate, 2 = extensive (>50%) peri-islet infiltrate, 3 = intraislet infiltrate, and 4 = extensive intraislet infiltrate (>80%) or total β-cell loss. The percentage of islets with each grade per pancreas was calculated by addition of the grades for the three sections. Individual insulitis scoring for each mouse was performed as previously described (22).

### Incidence of Diabetes and Insulitis

Diabetes onset was monitored by weekly measurement of urine glucose levels using Diastix (Bayer Diagnostics). Blood glucose levels were measured in mice with glycosuria using Advantage II Glucose strips (Roche). Animals displaying two consecutive blood glucose measurements of ≥ 15mmol/L were considered diabetic. For adoptive transfer of diabetes, 2 x10^7^ splenocytes from 13-17 week old pre-diabetic TIP-1 mice or control NOD mice were transferred (i.v.) into 9-12week old NOD Rag-/- recipients and diabetes development was monitored as above.

### Flow Cytometry

Antibodies used were anti-CD4 (RM4-5) conjugated to PerCpCy5.5, anti-CD3 (145-2C11) conjugated to FITC or anti-CD3 (500 A2) V500, anti-CD44 (1M7) conjugated to AlexaFlour700 (all BD Biosciences), anti-CD11c (N418), anti-B220 (RA3-6B2), anti-CD11b (M1/70), anti-F4/80 (BM8) conjugated to eFlour450 and anti-FoxP3 (FJK-16S) conjugated to APC (all eBiosciences), anti-CD8a (5H10) conjugated to Pacific Orange (Invitrogen) or anti-CD8a (53-6.7) conjugated to PE-Cy7, anti-CD62L (MEL-14) conjugated to APC-Cy7 (BD Biosciences). FoxP3 was stained intracellularly using FoxP3/Transcription Factor Fixation/Permeabilization kit (eBiosciences). Data were collected on an LSR Fortessa flow-cytometer (BD) and analyzed using FlowJo (Treestar) software.

### Tetramer and Magnetic Bead-Based Enrichment

The tetramer and magnetic bead-based enrichment method was previously described (25). I-Ag7 tetramers were obtained from NIH tetramer core facility (Emory University, Georgia, USA), Kd-tetramers were obtained from ImmunoID (Parkville, Victoria, Australia). To enrich insulin-specific CD4+ T cells single cell suspensions from peripheral lymphoid organs (PLO), (pooled spleen and non-draining lymph nodes), were stained with phycoerythrin (PE)-conjugated I-Ag7-INSB_10-23_ (HLVERLYLVCGGEG) tetramer for 1 hour at room temperature. The Ins B_10-23_ peptide in the I-Ag7-INSB_10-23_ tetramer has been mutated (Glutamic acid to Glycine (E-G) at position 20 and Arginine to Glycine (R-G) at position 21) to improve its binding to the I-Ag7 molecule, which allows for better detection of insulin-specific CD4+ T cells (26). Insulin-specific CD8+ T cells were enriched from pooled PLO by staining the cell suspensions with APC-conjugated H-2Kd- INSB_15-23_ (LYLVCGGEG) tetramer for 1 hour on ice. Hen Egg Lysozyme I-Ag7-HEL (AMKRHGLDNYRGYSL) tetramer or H-2Kd-TUM (KYQAVTTTL) were used as controls. Cells were then washed and stained with anti-PE or anti-APC microbeads (Miltenyi Biotec) followed by magnetic separation using an AutoMACSpro (Miltenyi Biotec) according to manufacturer’s instructions. IGRP_206-214_ specific CD8+ T cells (H2-Kd, VYLKTNVFL) were stained and enriched as previously described (27). The separated fractions were stained and analyzed by flow cytometry. Gating strategy for tetramer enrichment was as follows: single cells were gated on forward and side scatter, and dead cells excluded using propidium iodide. From the live cell population, CD11c-CD11b-B220-F4/80-CD3+ cells were gated as the T cell population for analysis. Further selection of CD4+ T cells or CD8+ T cells was followed by analysis of the insulin or IGRP tetramer positive population respectively.

### Insulin Autoantibody (IAA) Assay

A non-competitive IAA assay was performed in a 96 well ELISA format as previously described (28, 29). Briefly, an ELISA plate (Costar) was coated with or without human insulin (10 µg/ml, Actrapid, Novo Nordisk) overnight at 4°C. Wells were blocked with PBS containing 2% BSA for 2 hours and room-temperature and then probed with sera from 12-15 weeks old TIP mice, NOD or C57BL/6 mice (1:10 dilution) for 2 hours. Wells were washed 4 times and a biotinylated anti-mouse IgG1 (AbCam, 1:10000 dilution) antibody was added for 30 minutes. After washing, horse-radish-peroxidase conjugated streptavidin (BioLegend) was added for 15 minutes. The plate was washed five times, TMB substrate solution (BioLegend) was added and absorbance was measured at 450 nm using a Polarstar (BMG labtech) microplate reader. Each sample was run in duplicate and absorbance (450 nm) of test sample without plate bound insulin was subtracted from absorbance of test sample with plate bound insulin to calculate the actual absorbance value for each sample.

### Statistical Analysis

Statistical analysis was performed using GraphPad Prism 8 Software (GraphPad, San Diego, CA, USA). Pooled data are shown as dot-plots with individual mice and the mean ± SEM. Data were tested for normal distribution using D’Agostino-Pearson omnibus normality test or Shapiro-Wilks test. Comparisons between two groups were performed using two-tailed unpaired student *t*-tests. Multiple comparisons were performed using One-way ANOVA with Sidak’s post-hoc test. Survival curves were compared using Log-Rank (Mantel-Cox) test. Statistical significance was defined as *P* < 0.05.

## Results

### Conditional Expression of Proinsulin-1 in NOD Mice

**Figure 1:**
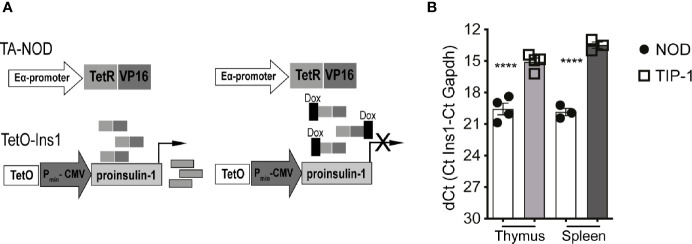
Conditional proinsulin 1 expression in TIP-1 mice **(A)** Scheme of generation of tetracycline regulated NOD.IEα-tTA (TA-NOD) and tetO-Ins1 dual transgenic mice referred to herein as TIP-1 mice. TA-NOD mice were crossed with tetO-Ins1 mice. Bi-transgenic animals constitutively express PIns1 in APCs **(B)** Quantitative RT-PCR was performed using Taqman probes for *Ins1* and *Gapdh* in thymic and splenic lysates of WT-NOD mice TIP-1 mice. Data represent dCT values (Mean ± SEM) from 2-3 independent experiments run in duplicate for each probe. ****P < 0.0001. Data compared using One-way ANOVA with Sidak’s multiple comparisons test.

To test whether inducing immune tolerance to proinsulin-1 (PIns1) influenced autoimmune diabetes we generated transgenic NOD mice to facilitate conditional expression of PIns1 in the antigen presenting cells (APCs). Reporter NOD mice expressing PIns1 under the control of the tetracycline-responsive CMV promoter (TetO-Ins1 mice) were bred with previously described driver NOD mice expressing TetR-VP16 tetracycline transactivator protein (tTA) under the control of IEα-MHC-II promoter referred to as TA-NOD mice (23). Bi-transgenic progeny referred to as TIP-1 (Tet Inducible PIns1) mice (**Figure 1A**) express Pins1 in the APCs, which can be turned-off upon doxycycline (Dox) treatment. Analysis of PIns1 expression in TIP-1 mice revealed that PIns1 transgene was expressed in the thymus and spleen as measured by RT-PCR (**Figure 1B**). After one week of Dox treatment, PIns1 expression dropped to baseline levels (**Figure S1**). Thus, PIns1 expression in TIP-1 mice was conditional, and tightly regulated.

### TIP-1 Mice Have Reduced Insulitis and Insulin Autoantibody Expression

**Figure 2:**
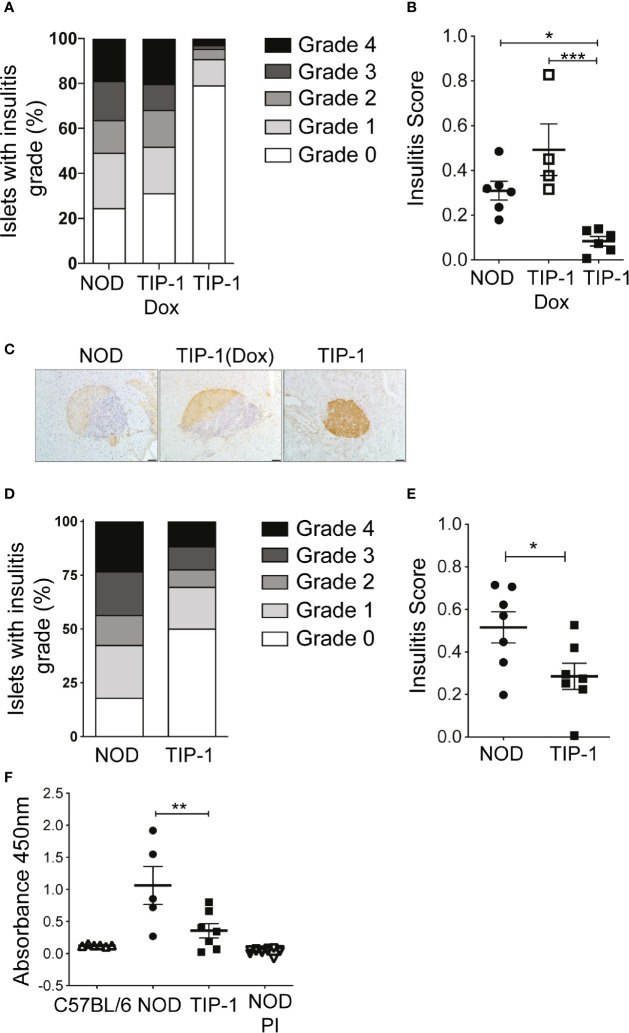
Insulitis and insulin autoantibodies (IAA) in TIP-1 mice **(A, B)** Histological grading and individual insulitis scores in NOD mice, Doxycycline treated and untreated TIP-1 mice at 12-14 weeks of age. **(C)** Representative images of islet histology from 12-14 week old NOD, TIP-1 and Dox treated TIP-1 mice, 200x magnification, scale bar 50 μm. **(D, E)** Histological grading and individual insulitis scores at 20-25 weeks of age in NOD mice and TIP-1 mice (n=4-7, > 60 islets scored per mouse). **(F)** Sera from 12-16 weeks old C57BL/6 mice, NOD mice, TIP-1 and NOD-PI mice were tested for the presence of insulin autoantibodies (IAA) by ELISA assay. Absorbance values at 450nm are plotted. Each symbol in the scatter plot represents data from individual animals. Data plotted as Mean ± SEM, ***P < 0.001,**P < 0.01, *P < 0.05. Data compared using One-way ANOVA with Sidak’s multiple comparisons test **(B, F)** and 2-tailed unpaired t-test **(E)**.

We recently reported that constitutive or temporal expression of PIns2 (PIns2) in the APCs limited to the perinatal period prevented insulitis and diabetes in NOD mice (22). To test whether PIns1 expression in the APCs influenced the progression of islet autoimmunity, we examined the immune infiltrate (insulitis) in the pancreata of TIP-1 mice expressing PIns1 continuously. At 12-14 weeks of age, insulitis was significantly reduced in TIP-1 mice expressing PIns1 compared to age matched NOD mice or TIP-1 mice fed dox to suppress PIns1 expression (**Figures 2A–C**). Analysis of pancreas histology from TIP-1 mice at 20-25 weeks of age revealed that approximately 50% of the islets examined were free of insulitis, whereas more than 80% of the islets examined from non-transgenic littermates were infiltrated (**Figures 2D, E** and **Table S1**), indicating that PIns1 expression in the APCs decreased but did not completely abolish development of insulitis, which progressed over time. Production of insulin autoantibodies (IAA) indicates spontaneous anti-insulin autoimmunity and IAA are frequently detected prior to diabetes onset in both humans and NOD mice (30, 31). We examined whether induced PIns1 expression in TIP-1 mice influenced B cell mediated humoral responses against insulin by measuring IAA in TIP-1 mice. IAA was significantly reduced in 12-15 weeks old TIP-1 mice as compared to age matched non-transgenic NOD mice (**Figure 2F**). Previously described PIns2 tolerant NOD-PI mice that are protected from diabetes and non-autoimmune prone C57BL/6 mice were used to set the baseline. Collectively, these results indicate that immune tolerance to PIns1 influenced progression of insulitis and reduced the development of IAA.

### PIns1 Overexpression Partially Suppresses Spontaneous Diabetes in NOD Mice

**Figure 3:**
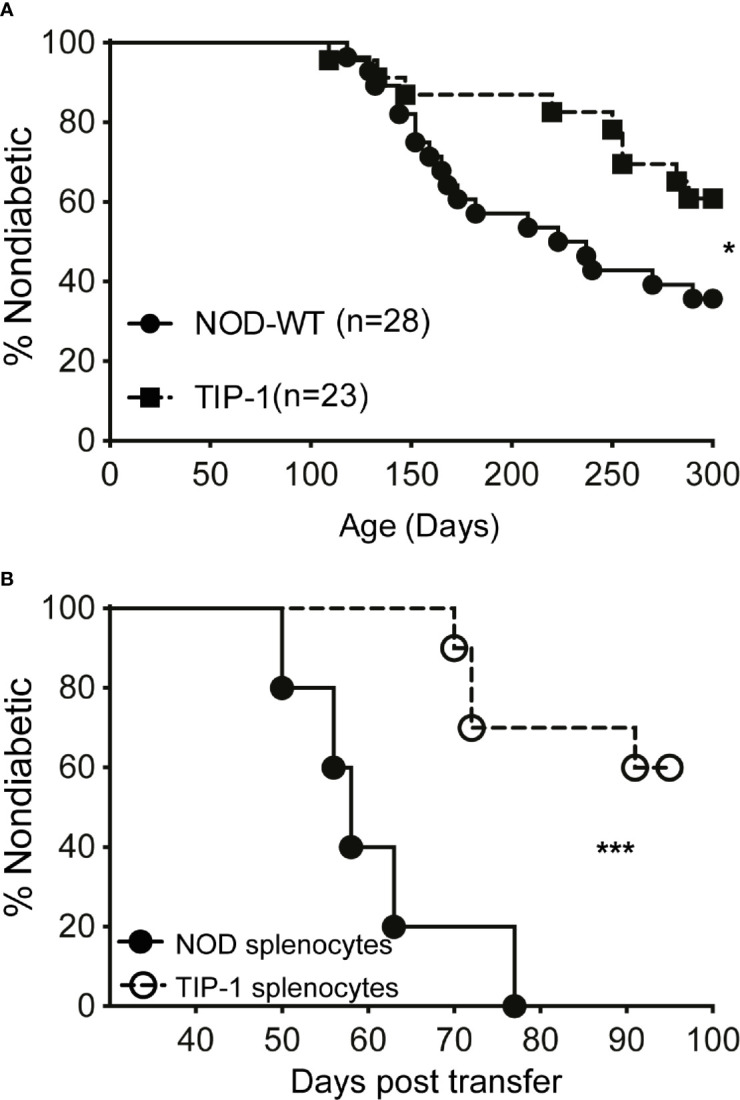
Spontaneous diabetes incidence in TIP-1 mice **(A)** Incidence of spontaneous diabetes in female TIP-1 mice and non-transgenic littermates until 300 days of age. Numbers in parentheses indicate the number of mice analyzed. **(B)** Incidence of diabetes following transfer of splenocytes (2 x 10^7^ cells/recipient) from 13-17 weeks old non-diabetic TIP-1 mice or NOD mice into 8-9 weeks old NOD.Rag1^-/-^ recipients (n > 5 each). *P < 0.05; ***P < 0.0001. Survival curves were compared using log-rank (Mantel-Cox) test.

Reduced insulitis and IAA suggest that diabetes development may be altered in TIP-1 mice. A cohort of female TIP-1 mice expressing PIns1 continuously and control NOD mice were observed for incidence of spontaneous diabetes. TIP-1 mice developed diabetes but at a significantly reduced incidence compared to non-transgenic control NOD mice. By 300 days of age 40% of TIP-1 mice and 65% of the control mice developed diabetes (**Figure 3A**). In addition, we investigated whether PIns1 expression in TIP-1 mice influenced the pathogenic potential of effector T cells. Splenocytes from 15-18 weeks old TIP-1 mice with ongoing expression of PIns1 and age matched control NOD mice were transferred into NOD.Rag1 -/- recipients. All recipient mice receiving control splenocytes developed diabetes between 50-70 days post-transfer, whereas only 2 out of 6 (33%) animals that received splenocytes from TIP-1 mice developed diabetes 70-90 days post-transfer (**Figure 3B**). Taken together these results suggest that overexpression of PIns1 in APCs is able to partially dampen immune responses against insulin and reduce diabetes incidence in NOD mice.

### Proinsulin-Specific Tolerance in TIP-1 Mice

**Figure 4:**
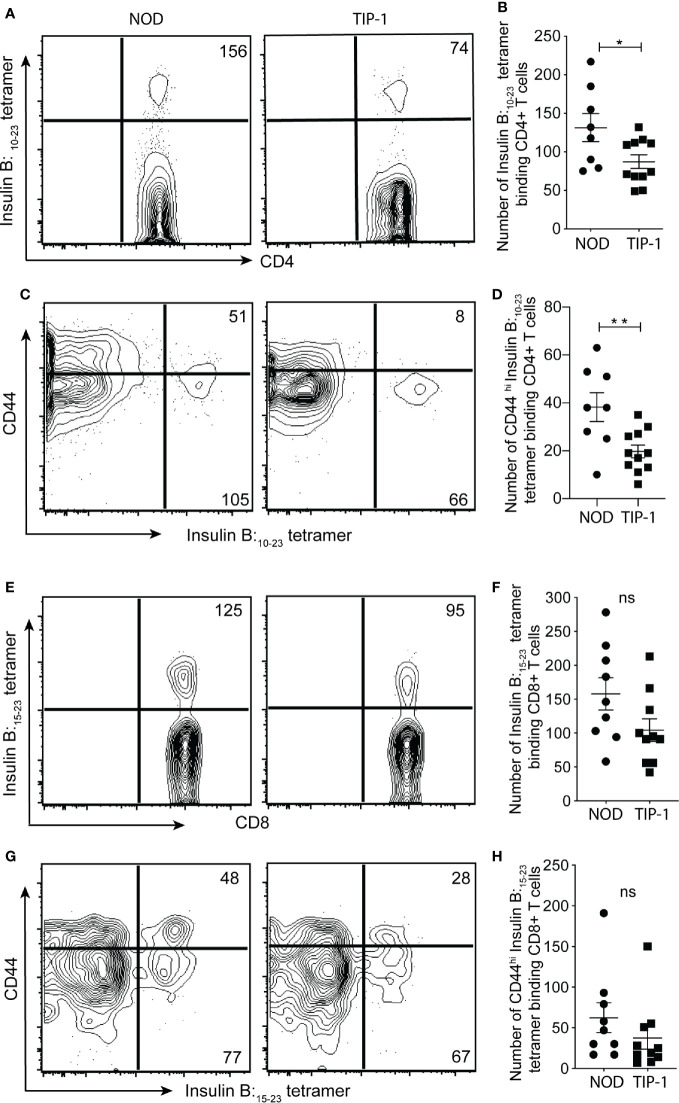
Immune tolerance to insulin specific T cells in TIP-1 mice Insulin B:_10-23_-specific CD4+ T cells or Insulin B:_15-23_-specific CD8+ T cells were stained with respective tetramers and enriched from pooled peripheral lymphoid organs (PLO) of 20-25 weeks old TIP-1 mice and NOD mice using magnetic beads and enumerated by flow-cytometry. Representative FACS plots **(A, C, E, G)** and enumeration of insulin tetramer+ CD4+ T **(B)** cells, insulin tetramer+ CD8+ T cells **(F)**, CD44^hi^ Insulin tetramer + CD4+ T cells **(D)** and CD44^hi^ Insulin tetramer + CD8+ T cells **(H)** in TIP-1 and NOD mice. Values in the FACS plots indicate absolute number of tetramer binding cells. Each symbol in the scatter plots (Mean ± SEM) represents data from an individual mouse. **P < 0.01, *P < 0.05, ns= not significant. Data compared using 2-tailed unpaired t-test.

The partial protection from insulitis and diabetes in TIP-1 mice expressing PIns1 in the APCs could be due to immune tolerance to PIns1 epitopes. To demonstrate tolerance to PIns1, we enumerated the frequency of Insulin B _9-23_ reactive CD4+ T cells and Insulin B _15-23_ reactive CD8+ T cells in the peripheral lymphoid organs (PLO) (pooled spleen and non-draining lymph nodes) of 20-25 weeks old non-diabetic TIP-1 mice and age matched control mice using respective I-A (g^7^) and K^d^ tetramers. There was a significant reduction in the absolute number of insulin B:_9-23_ specific CD4+ T cells binding to insulin B:_10-23_/I-A (g^7^) tetramer (26) and the antigen-experienced CD44^hi^ subset of insulin B:_9-23_ specific CD4+ T cells in TIP-1 mice (**Figures 4A–D**). The absolute number of CD8+T cells recognizing insulin B:_15-23_ epitope (32) as well as the number of antigen-experienced CD44^hi^ subset of insulin B:_15-23_ specific CD8+ T cells were comparable in both TIP-1 mice and controls (**Figures 4E–H**). While the significant reduction of insulin-specific CD4+ and CD8+ T cells in TIP-1 is suggestive of deletional tolerance, it is possible that transgenic antigen expression in APCs may induce regulatory T cells (Tregs) that confer dominant tolerance and prevent diabetes in TIP-1 mice. We examined the expression of Foxp3 on insulin B:_9-23_ specific CD4+ T cells in PLO of TIP-1 mice and non-transgenic controls and did not observe any significant differences (**Figure S2A, B**), In addition we examined the frequency of Foxp3+ CD4+ Tregs in the thymus and pancreatic lymph node (PLN). The proportion of Tregs was similar in both TIP-1 and control mice (**Figure S2C, D**).Taken together our data indicate that ectopic PIns1 expression induces deletion of cognate CD4+ T cells, but does not induce antigen specific Tregs. The few remaining insulin reactive CD4+T cells could not be activated by the expressed antigen, whereas the low-affinity insulin B:_15-23_ reactive CD8+ T cells (33) are not influenced by transgenic PIns1 expression.

### Downstream Responses to IGRP Are Delayed in TIP-1 Mice

**Figure 5:**
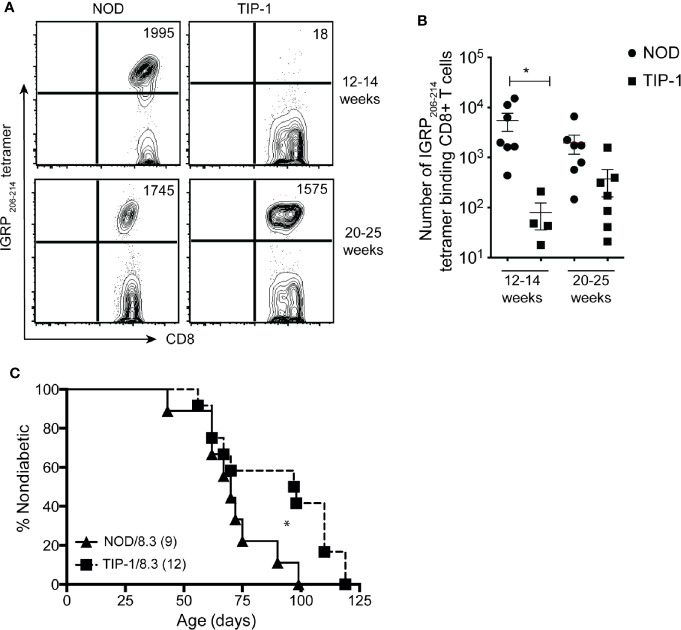
Enumeration of IGRP specific CD8+ T cells in TIP-1 mice IGRP _206-214_-specific CD8+ T cells were stained with Kd- IGRP tetramer and enriched from pooled peripheral lymphoid organs (PLO) of 12-14 weeks and 20-25 weeks old NOD mice and TIP-1 mice. Representative FACS plots **(A)** and quantification **(B)** of absolute number of IGRP _206-214_ tetramer + CD8+ T cells at indicated ages. Values in the FACS plots indicate absolute number of tetramer binding cells. Each symbol in the scatter plots (Mean ± SEM) represents data from an individual mouse. *P<0.05, data in **(B)** compared using one-way ANOVA with Sidak’s multiple comparisons test. **(C)** Incidence of spontaneous diabetes in female TIP-1/8.3 mice and NOD 8.3 littermates. Numbers in parentheses indicate the number of mice analyzed. *P < 0.05. Survival curves were compared using log-rank (Mantel-Cox) test.

Previous work from our group has demonstrated that autoreactive responses to islet-specific glucose-6-phosphatase catalytic subunit-related protein (IGRP) are dependent upon immune response to PIns2 (1). To investigate if tolerance to PIns1 influenced the immune response to IGRP we examined the frequency of pathogenic IGRP _206-214_ reactive CD8+ T cells in TIP-1 mice. The number of IGRP _206-214_ specific CD8+ T cells was significantly reduced in 12-14 weeks old TIP-1 mice expressing PIns1 as compared to age matched controls. However, the frequency of IGRP _206-214_ specific CD8+ T cells in TIP-1 mice expressing PIns1 did not differ from age-matched controls at 20-25 weeks of age (**Figures 5A, B**). This indicates that tolerance to PIns1 delays but does not prevent the spreading of immune responses to downstream antigen IGRP.

### Immune Response to Proinsulin-1 Is Not Required for Diabetes in NOD 8.3 Mice

Autoreactivity to PIns2 is required for diabetes development in NOD 8.3 mice that have a pre-existing repertoire of IGRP specific T cells (34). Since we observed reduced frequency of IGRP reactive CD8+ T cells in 12-14 weeks old TIP-1 mice, we wished to know if immune responses to PIns1 were necessary for diabetes development in NOD 8.3 mice. TIP-1 mice were crossed with NOD 8.3 mice to generate offspring that were TIP-1/NOD8.3 double transgenic or NOD 8.3 transgenic alone. TIP-1/8.3 mice developed diabetes with significantly delayed kinetics (median survival 97 days) compared to NOD8.3 mice (median survival 70 days) but all mice eventually developed disease (**Figure 5C**). The frequency of insulin specific T cells is very low even in NOD mice and with a skewed T cell repertoire in NOD 8.3 transgenic mice it is not possible to detect any insulin specific T cells. We were unable to detect insulin-specific T cells in TIP-1/8.3 mice (data not shown). Therefore, tolerance to PIns1 significantly delays but does not prevent diabetes development in NOD 8.3 mice.

## Discussion

In this study we generated transgenic NOD mice to induce PIns1 expression in the APCs and examined the impact of antigen specific tolerance on autoimmune diabetes. The main findings of this study are 1) TIP-1 mice expressing PIns1 in the APCs show significantly reduced incidence of diabetes, which is associated with reduced insulitis and insulin autoantibody (IAA) expression. 2) Proinsulin specific CD4+ T cells are detectable in TIP-1 mice at a reduced frequency and are not activated. 3) Immune responses to downstream antigen IGRP are delayed but not absent in TIP-1 mice.

Given the high degree of homology between proinsulin 1 and 2 proteins, especially in the immunodominant insulin B chain epitope Ins B:_9-23_ we expected to achieve robust protection from diabetes onset in TIP-1 mice, similar to previously described proinsulin-2 tolerant NOD mice (35); however, the partial protection from insulitis and diabetes observed in TIP-1 mice points to the existence of distinct pathogenic peptide epitopes in the PIns2 protein that can precipitate autoimmunity in NOD mice. A previous study characterizing immunogenic epitopes in NOD mice reported existence of multiple epitopes on both PIns1 and PIns2 molecules recognized by CD4+T cells (18). Importantly, epitopes outside of the highly homologous Ins B:_9-23_ peptide were identified in the leader and A chain sequences of PIns2 molecule. Thus, it is likely that PIns2 reactive T-cells recognizing these unique epitopes may induce islet destruction and subsequent diabetes onset in TIP-1 mice.

Our data complement the previous observations that reported detection of PIns1 reactive T cells (18, 19) in NOD mice. While the previous studies did not directly demonstrate the role of PIns1-reactive T cells in spontaneous disease, the significant reduction in diabetes incidence in TIP-1 mice suggests that PIns1 specific T cells participate in autoimmune destruction of beta cells. On the other hand, development of IAA and diabetes in TIP-1 mice may be related to ongoing immune responses to PIns2 epitopes.

A drawback of our study is that we have analyzed a single transgenic founder line expressing PIns1 in the APCs. Varying levels of transgenic insulin expression in the thymus may influence the diabetes progression in NOD mice. PIns2 levels were 7-fold higher in the spleen (~140 pmol/L) as compared to thymus (~20pmol/L) in the partially protected Pins2 tolerant mice previously described by Jaeckel et al. (12), whereas in the recently described TIP mice with robust protection from autoimmune diabetes upon conditional PIns2 expression in APCs, the level of thymic Pins2 expression (100pmol/L) was 5-fold more compared to peripheral tissues(20pmol/L) (22). TIP-1 mice may have relatively reduced transgenic expression of PIns1 in the thymic APCs as compared to transgenic PIns2 expression in the previously described TIP mice, thus imparting incomplete protection from autoimmune diabetes. Chentoufi and Polychronakos previously reported that Ins2 is expressed at a level more than 3-fold higher than Ins1 in the thymus of NOD mice (9). In TIP-1 mice analyzed here, induction of PIns1 results in approximately 5-fold higher expression as compared to non-transgenic NOD mice or uninduced TIP-1 mice. Moreover, protection from insulitis in TIP-1 mice is associated with the expression of PIns1 transgene, as TIP-1 mice fed doxycycline to suppress PIns1 expression develop islet infiltration comparable to non-transgenic controls indicating that ectopic PIns1 expression in APCs influences anti-islet immunity.

Does the reduction in the incidence of spontaneous diabetes in TIP-1 mice correlate with deletion of PIns1 specific T cells? Insulin B:_10-23_ and Insulin B:_15-23_ specific tetramers used in our study are likely to detect both PIns1 and 2 reactive CD4+ and CD8 +T cells, due to the invariant nature of the Insulin B:_9-23_ peptide between the two isoforms. Immune responses to Insulin B:_9-23_ epitope are required for both priming and effector phase of islet autoimmunity in NOD mice. Moreover, Insulin B:_9-23_ primed CD4+ T cells are able to induce islet autoimmunity evidenced by IAA production (36). The significant reduction in absolute number of Insulin B:_9-23_ tetramer binding CD4+T cells, and the antigen-experienced subset of tetramer binding CD4+ T cells, coupled with reduced IAA production in TIP-1 mice is suggestive of antigen-specific tolerance. While Tregs are an important tolerance mechanism, we did not find any evidence to suggest that the partial protection from diabetes in TIP-1mice is due to antigen-specific Tregs. We are currently unable to conclude whether central or peripheral tolerance mechanisms regulate the insulin specific T cells in TIP-1 mice; however, future studies with ectopic antigen expression induced after the exit of antigen-specific T cells from the thymus may resolve this question.

Autoimmunity to insulin determines immune responses to other downstream antigens such as IGRP (1). IGRP _206-214_ reactive CD8+ T cells were reduced in TIP-1 mice at 12-14 weeks; but ongoing tolerance to PIns1 did not prevent development of diabetes onset in TIP-1/8.3 mice. The precursor frequency of IGRP reactive CD8+ T cells is low in NOD mice (27), and the residual immune response to PIns2 in TIP-1 mice may be reduced as compared to control mice. The reduced CD4+ T cell help possibly accounts for the delayed expansion of IGRP specific T cells seen in TIP-1 mice. However, the residual immune response to PIns2 in TIP-1/8.3 mice with a pre-existing repertoire of IGRP specific T cells may be sufficient to help IGRP specific CD8+ T cells to mediate beta-cell destruction.

In summary, we find that immune tolerance to PIns1, whilst partly protective, is not sufficient to prevent spontaneous diabetes in NOD mice. Our data clarifies the role of PIns1 in the pathogenesis of autoimmune diabetes in NOD mice and extends the previously established role of PIns1 in autoimmune diabetes. The experimental model we have presented here, with its conditional gene-expression system, has the potential to delineate whether antigen-specific interventions can induce immune tolerance after islet autoimmunity is well established. Understanding this is important for development of strategies to induce antigen-specific tolerance clinically in people with stage 1 or 2 type 1 diabetes (37).

## Data Availability Statement

The original contributions presented in the study are included in the article/**Supplementary Material**. Further inquiries can be directed to the corresponding author.

## Ethics Statement

The animal study was reviewed and approved by Animal Ethics Committee, St Vincents Hospital, Melbourne.

## Author Contributions

GJ performed experiments, analyzed data, and wrote the manuscript. CS, JC, C-TK, and EP performed experiments and analyzed data. HT, BK, and TK designed the study, analyzed data, and wrote the manuscript. BK and TK supervised the study. All authors contributed to the article and approved the submitted version.

## Funding

This work was funded by the National Health and Medical Research Council of Australia (GNT1037321 and GNT1150425) and fellowship from Juvenile Diabetes Research Foundation (BK). The St Vincent’s Institute receives support from the Operational Infrastructure Support Scheme of the Government of Victoria.

## Conflict of Interest

The authors declare that the research was conducted in the absence of any commercial or financial relationships that could be construed as a potential conflict of interest.

## References

[1] Krishnamurthy B Dudek NL McKenzie MD Purcell AW Brooks AG Gellert S. Responses against islet antigens in NOD mice are prevented by tolerance to proinsulin but not IGRP. J Clin Invest (2006) 116(12):3258–65. 10.1172/JCI29602 PMC1679712 17143333

[2] Mannering SI Pathiraja V Kay TW. The case for an autoimmune aetiology of type 1 diabetes. Clin Exp Immunol (2016) 183(1):8–15. 10.1111/cei.12699 26313217 PMC4687512

[3] Lucassen AM Julier C Beressi J-P Boitard C Froguel P Lathrop M. Susceptibility to insulin dependent diabetes mellitus maps to a 4.1 kb segment of DNA spanning the insulin gene and associated VNTR. Nat Genet (1993) 4(3):305–10. 10.1038/ng0793-305 8358440

[4] Pugliese A Zeller M Fernandez A Jr. Zalcberg LJ Bartlett RJ Ricordi C. The insulin gene is transcribed in the human thymus and transcription levels correlated with allelic variation at the INS VNTR-IDDM2 susceptibility locus for type 1 diabetes. Nat Genet (1997) 15(3):293–7. 10.1038/ng0397-293 9054945

[5] Vafiadis P Bennett ST Todd JA Nadeau J Grabs R Goodyer CG. Insulin expression in human thymus is modulated by INS VNTR alleles at the IDDM2 locus. Nat Genet (1997) 15(3):289–92. 10.1038/ng0397-289 9054944

[6] Wentworth BM Schaefer IM Villa-Komaroff L Chirgwin JM. Characterization of the two nonallelic genes encoding mouse preproinsulin. J Mol Evol (1986) 23(4):305–12. 10.1007/BF02100639 3104603

[7] Deltour L Leduque P Blume N Madsen O Dubois P Jami J. Differential expression of the two nonallelic proinsulin genes in the developing mouse embryo. Proc Natl Acad Sci U S A (1993) 90(2):527–31. 10.1073/pnas.90.2.527 PMC45696 8421685

[8] Heath VL Moore NC Parnell SM Mason DW. Intrathymic expression of genes involved in organ specific autoimmune disease. J Autoimmun (1998) 11(4):309–18. 10.1006/jaut.1998.0210 9776708

[9] Chentoufi AA Polychronakos C. Insulin expression levels in the thymus modulate insulin-specific autoreactive T-cell tolerance: the mechanism by which the IDDM2 locus may predispose to diabetes. Diabetes (2002) 51(5):1383–90. 10.2337/diabetes.51.5.1383 11978634

[10] Wegmann DR Norbury-Glaser M Daniel D. Insulin-specific T cells are a predominant component of islet infiltrates in pre-diabetic NOD mice. Eur J Immunol (1994) 24(8):1853–7. 10.1002/eji.1830240820 8056042

[11] Nakayama M Abiru N Moriyama H Babya N Liu E Miao D. Prime role for an insulin epitope in the development of type 1 diabetes in NOD mice. Nature (2005) 435(7039):220–3. 10.1038/nature03523 PMC1364531 15889095

[12] Jaeckel E Lipes MA von Boehmer H. Recessive tolerance to preproinsulin 2 reduces but does not abolish type 1 diabetes. Nat Immunol (2004) 5(10):1028–35. 10.1038/ni1120 15378058

[13] Daniel D Wegmann DR. Protection of nonobese diabetic mice from diabetes by intranasal or subcutaneous administration of insulin peptide B-(9-23). Proc Natl Acad Sci U S A (1996) 93(2):956–60. 10.1073/pnas.93.2.956 PMC40166 8570667

[14] Devendra D Paronen J Moriyama H Miao D Eisenbarth GS Liu E. Differential immune response to B:9-23 insulin 1 and insulin 2 peptides in animal models of type 1 diabetes. J Autoimmun (2004) 23(1):17–26. 10.1016/j.jaut.2004.03.008 15236749

[15] Thebault-Baumont K Dubois-Laforge D Krief P Briand J-P Halbout P Vallon-Geoffroy K. Acceleration of type 1 diabetes mellitus in proinsulin 2-deficient NOD mice. J Clin Invest (2003) 111(6):851–7. 10.1172/JCI16584 PMC153768 12639991

[16] Moriyama H Abiru N Paronen J Sikora K Liu E Miao D. Evidence for a primary islet autoantigen (preproinsulin 1) for insulitis and diabetes in the nonobese diabetic mouse. Proc Natl Acad Sci U S A (2003) 100(18):10376–81. 10.1073/pnas.1834450100 PMC193569 12925730

[17] Elso CM Scott NA Mariana L Masterman EI Sutherland APR Thomas HE. Replacing murine insulin 1 with human insulin protects NOD mice from diabetes. PLoS One (2019) 14(12):e0225021. 10.1371/journal.pone.0225021 31821343 PMC6903741

[18] Halbout P Briand J-P Becourt C Muller S Boitard C. T cell response to preproinsulin I and II in the nonobese diabetic mouse. J Immunol (2002) 169(5):2436–43. 10.4049/jimmunol.169.5.2436 12193712

[19] Levisetti MG Lewis DM Suri A Unanue ER. Weak proinsulin peptide-major histocompatibility complexes are targeted in autoimmune diabetes in mice. Diabetes (2008) 57(7):1852–60. 10.2337/db08-0068 PMC2453633 18398138

[20] Faideau B Lotton C Lucas B Tardivel I Elliott JF Boitard C. Tolerance to proinsulin-2 is due to radioresistant thymic cells. J Immunol (2006) 177(1):53–60. 10.4049/jimmunol.177.1.53 16785498

[21] Fan Y Rudert WA Grupillo M He J Sisino G Trucco M. Thymus-specific deletion of insulin induces autoimmune diabetes. EMBO J (2009) 28(18):2812–24. 10.1038/emboj.2009.212 PMC2750011 19680229

[22] Jhala G Chee J Trivedi PM Selck C Gurzov EN Graham KL. Perinatal tolerance to proinsulin is sufficient to prevent autoimmune diabetes. JCI Insight (2016) 1(10):e86065. 10.1172/jci.insight.86065 27699217 PMC5033903

[23] Guerau-de-Arellano M Martinic M Benoist C Mathis D. Neonatal tolerance revisited: a perinatal window for Aire control of autoimmunity. J Exp Med (2009) 206(6):1245–52. 10.1084/jem.20090300 PMC2715060 19487417

[24] Verdaguer J Schmidt D Amrani A Anderson B Averill N Santamaria P. Spontaneous autoimmune diabetes in monoclonal T cell nonobese diabetic mice. J Exp Med (1997) 186(10):1663–76. 10.1084/jem.186.10.1663 PMC2199139 9362527

[25] Moon JJ Chu HH Hataye J Pagan AJ Pepper M McLachlan JB. Tracking epitope-specific T cells. Nat Protoc (2009) 4(4):565–81. 10.1038/nprot.2009.9 PMC3517879 19373228

[26] Crawford F Stadinski B Jin N Michels A Nakayama M Pratt P. Specificity and detection of insulin-reactive CD4+ T cells in type 1 diabetes in the nonobese diabetic (NOD) mouse. Proc Natl Acad Sci U S A (2011) 108(40):16729–34. 10.1073/pnas.1113954108 PMC3189014 21949373

[27] Chee J Ko H-J Skowera A Jhala G Catterall T Graham KL. Effector-memory T cells develop in islets and report islet pathology in type 1 diabetes. J Immunol (2014) 192(2):572–80. 10.4049/jimmunol.1302100 24337380

[28] Babaya N Liu E Miao D Li M Yu L Eisenbarth GS. Murine high specificity/sensitivity competitive europium insulin autoantibody assay. Diabetes Technol Ther (2009) 11(4):227–33. 10.1089/dia.2008.0072 PMC2903340 19344197

[29] Daniel C Weigmann B Bronson R von Boehmer H. Prevention of type 1 diabetes in mice by tolerogenic vaccination with a strong agonist insulin mimetope. J Exp Med (2011) 208(7):1501–10. 10.1084/jem.20110574 PMC3135372 21690251

[30] Yu L Robles DT Abiru N Kaur P Rewers M Kelemen GS. Early expression of antiinsulin autoantibodies of humans and the NOD mouse: evidence for early determination of subsequent diabetes. Proc Natl Acad Sci U S A (2000) 97(4):1701–6. 10.1073/pnas.040556697 PMC26499 10677521

[31] Ziegler AG Rewers M Simell O Simell T Lempainen J Steck A. Seroconversion to multiple islet autoantibodies and risk of progression to diabetes in children. JAMA (2013) 309(23):2473–9. 10.1001/jama.2013.6285 PMC4878912 23780460

[32] Wong FS Karttunen J Dumont C Wen L Visintin I Pilip IM. Identification of an MHC class I-restricted autoantigen in type 1 diabetes by screening an organ-specific cDNA library. Nat Med (1999) 5(9):1026–31. 10.1038/12465 10470079

[33] Wong FS Siew LK Scott G Thomas IJ Chapman S Viret C. Activation of insulin-reactive CD8 T-cells for development of autoimmune diabetes. Diabetes (2009) 58(5):1156–64. 10.2337/db08-0800 PMC2671054 19208910

[34] Krishnamurthy B Mariana L Gellert SA Colman PG Harrison LC Lew AM. Autoimmunity to both proinsulin and IGRP is required for diabetes in nonobese diabetic 8.3 TCR transgenic mice. J Immunol (2008) 180(7):4458–64. 10.4049/jimmunol.180.7.4458 18354167

[35] French MB Allison J Cram DS Thomas HE Dempsey-Collier M Silva A. Transgenic expression of mouse proinsulin II prevents diabetes in nonobese diabetic mice. Diabetes (1997) 46(1):34–9. 10.2337/diabetes.46.1.34 8971078

[36] Nakayama M Beilke JN Jasinski JM Kobayashi M Miao D Li M. Priming and effector dependence on insulin B:9-23 peptide in NOD islet autoimmunity. J Clin Invest (2007) 117(7):1835–43. 10.1172/JCI31368 PMC1904318 17607359

[37] Insel RA Dunne JL Atkinson MA Chiang JL Dabelea D Gottlieb PA. Staging presymptomatic type 1 diabetes: a scientific statement of JDRF, the Endocrine Society, and the American Diabetes Association. Diabetes Care (2015) 38(10):1964–74. 10.2337/dc15-1419 PMC5321245 26404926

