# Supplementary material for: Tolerance to Proinsulin-1 Reduces Autoimmune Diabetes in NOD Mice
Source: Front Immunol. 2021 Mar 25;12:645817. doi: 10.3389/fimmu.2021.645817 (PMC8027244; doi:10.3389/fimmu.2021.645817)
Supplement: Supplementary file 1 [file DataSheet_1.docx]

**Supplementary table**

**Table S1: Percentage insulitis in each histological grade**

|  | **12-14 weeks of age** | | | **20-25 weeks of age** | |
| --- | --- | --- | --- | --- | --- |
|  | **NOD** | **TIP-1-Dox** | **TIP-1** | **NOD** | **TIP-1** |
| **Grade 0** | 24.60 | 31.19 | 79.12 | 18.00 | 50.17 |
| **Grade 1** | 24.60 | 20.69 | 11.77 | 24.59 | 19.34 |
| **Grade 2** | 14.50 | 16.32 | 4.49 | 13.87 | 8.20 |
| **Grade 3** | 17.40 | 11.60 | 1.69 | 20.22 | 10.72 |
| **Grade 4** | 18.80 | 20.11 | 2.90 | 23.32 | 11.57 |

**Supplementary figures**


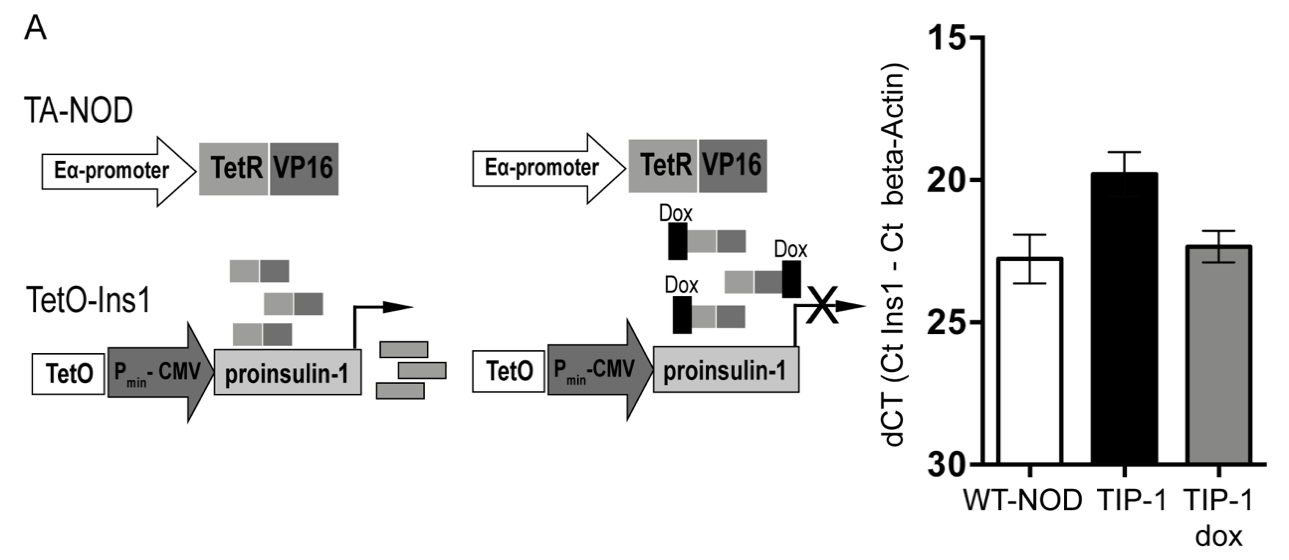


**Supplementary figure 1: Conditional proinsulin-1 expression in TIP-1 mice**

TIP-1 mice constitutively express PIns1 in APCs and were fed doxycycline (Dox) (2mg/ml) via drinking water to suppress transgene expression. Quantitative RT-PCR was performed using Taqman probes for *Ins1* and *Actb* on thymic lysates of WT-NOD mice TIP-1 mice. Data represent dCT values (Mean±SEM) from 2-3 independent experiments run in duplicate for each probe.


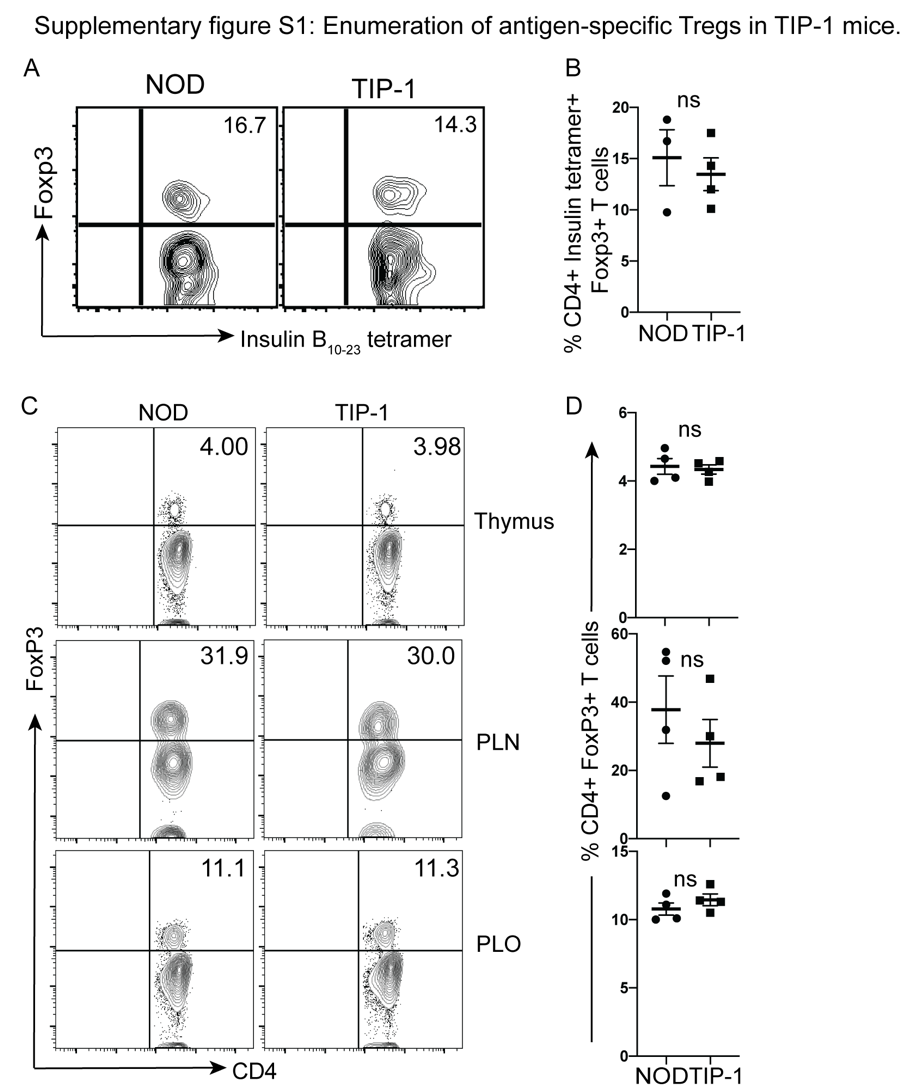


**Supplementary figure 2: Enumeration of Tregs in TIP-1 mice.**

Insulin_10–23_ tetramer^+^ CD4^+^ T cells were enriched from pooled spleens and non-draining lymph nodes (PLO) of TIP mice and control NOD mice, and intracellular FoxP3 expression was analyzed by flow cytometry. (A) Representative FACS plots showing FoxP3 expression on insulin tetramer^+^ cells and (B) frequency of FoxP3+ Insulin_10–23_ tetramer^+^ CD4^+^ T cells in the indicated mice. Frequency of FoxP3+ CD4+ Tregs was also determined in thymus, PLN and PLO of NOD and TIP-1 mice. (C) Representative FACS plots and (D) frequency of Tregs in the indicated tissues. Values in the top right quadrant of the FACS plots indicate percentage. Each symbol in the scatter plots (mean ± SEM) represents data from an individual mouse. Groups compared using 2-tailed unpaired t test. ns= not significant.
